# Comprehensive Multi-Omics Analysis of Muscle Tissue Alterations in Male *Macrobrachium rosenbergii* Induced by Frequent Mating

**DOI:** 10.3390/ijms26093995

**Published:** 2025-04-23

**Authors:** Yunpeng Fan, Qiang Gao, Haihua Cheng, Xilian Li, Huwei Yuan, Xue Cai, Lin Tang, Xiudan Yuan, Guangjing Zhang, Haiqi Zhang

**Affiliations:** 1 Agriculture Ministry Key Laboratory of Healthy Freshwater Aquaculture, Key Laboratory of Fish Health and Nutrition of Zhejiang Province, Zhejiang Institute of Freshwater Fisheries, Huzhou 313001, China; yunpengfan1994@163.com (Y.F.); gaoqiang@zjfish.com.cn (Q.G.); m130101022@163.com (H.C.); lixilian@126.com (X.L.); yuanhuwe@163.com (H.Y.); cx2423465312@163.com (X.C.); 221122270111@zjut.edu.cn (L.T.); 2 School of Marine Science, Ningbo University, Ningbo 315211, China; 3 Hunan Fisheries Science Institute, Changsha 410153, China; yuanxd2024@163.com; 4 College of Life Sciences, Hunan Normal University, Changsha 410081, China; m18737367413@163.com

**Keywords:** male *Macrobrachium rosenbergii*, frequent mating, omics, muscle, energy metabolism

## Abstract

During the breeding process of *Macrobrachium rosenbergii*, a male-to-female ratio of 1:3 or higher is typically adopted, so as a result, the quality of the male broodstock significantly influences the quality of the offspring. We observed that overused males exhibited notable changes in body color, particularly in the tail fan region, which turned orange or red due to the excessive accumulation of astaxanthin in the muscles and exoskeleton. Frequent mating also led to a significant decrease in male body weight, with histological analysis revealing disorganized muscle fiber patterns and increased tissue damage. To investigate the molecular mechanisms underlying these physiological changes, we performed transcriptomic and metabolomic analyses of muscle tissues. A total of 1069 differentially expressed genes (DEGs), 540 differentially expressed proteins (DEPs), and 385 differentially expressed metabolites (DEMs) were identified. Pathway analysis revealed that the DEGs were significantly enriched in pathways related to energy metabolism and degenerative diseases, while the DEMs were notably associated with cancer metabolism, signal transduction, substance transport, energy metabolism, nucleic acid metabolism, neurotransmission, immune response, and metabolic diseases. Proteome analysis showed that proteins and lipids were involved in muscle energy supply. These findings suggest that male *M. rosenbergii* upregulate energy metabolism in muscles to cope with frequent mating stress, but this adaptation leads to physiological damage. This study provides valuable insights for optimizing male broodstock selection and mating frequency in *M. rosenbergii* breeding practices.

## 1. Introduction

The giant freshwater prawn, *Macrobrachium rosenbergii*, an economically significant species, is widely cultured due to its rapid growth rate, excellent meat quality, and high nutritional value, making it an essential component of global aquaculture [1]. In natural ecosystems, the reproductive behavior of *M. rosenbergii* is characterized by significant seasonality and high frequency [2,3]. During the breeding season, male *M. rosenbergii* exhibit a strong mating drive and frequent mating behaviors [4]. Mating is an energy-intensive physiological process involving complex neuroendocrine regulation and muscle activity [5]. Studies have shown that mating behavior not only directly influences the reproductive success of male animals but may also have profound effects on growth, energy reserves, and survival capabilities [6,7]. For instance, in insects, fish, and mammals, frequent mating has been shown to induce significant changes in energy metabolism, potentially leading to muscle fatigue and growth suppression [7,8,9]. However, the impact of frequent mating on the muscle energy metabolism and overall physiological performance in *M. rosenbergii* remains underexplored.

The muscle tissue of *M. rosenbergii* is a key site for energy metabolism and plays a critical role in determining meat quality. Muscle energy metabolism involves several essential pathways, including glycolysis, the tricarboxylic acid (TCA) cycle, oxidative phosphorylation, and fatty acid metabolism [10]. The proper functioning of these pathways is crucial for maintaining muscle structure and function. However, it remains unclear whether frequent mating significantly impacts the muscle energy metabolism of male M. rosenbergii, and if so, what the underlying molecular mechanisms might be. Understanding how mating behavior affects muscle energy metabolism in *M. rosenbergii* is crucial from both ecological and aquaculture perspectives. In the wild, males must balance mating activities with energy reserves to ensure reproductive success and survival [4]. In aquaculture, frequent mating may negatively impact growth performance and meat quality, thereby affecting farming efficiency. Typically, breeders rely on experience to cull male prawns in poor condition, with key criteria including body color, shell luster, the morphology of the second pereiopod, and activity levels within the pond [11,12,13]. To address these issues, an integrative approach that combines multi-omics analyses with phenotypic observations is essential.

The biological activities of living organisms are characterized by a high degree of complexity. While genes carry genetic information, the transcriptional, protein, and metabolic levels are influenced by a multitude of factors, rendering the conclusions drawn from single-omics studies potentially unreliable [14,15]. Integrative multi-omics analysis has emerged as a significant tool in the realm of systems biology research, driven by the rapid advancements in biotechnology and the continuous reduction in costs [16]. The integration of biological phenotypes with multi-omics analyses facilitates the exploration of connections between different molecular levels and phenotypes, thereby enabling the systematic elucidation of the functions and regulatory mechanisms of biomolecules [17].

In this study, we aim to investigate the physiological and molecular impacts of frequent mating on male *M. rosenbergii* using a multi-omics approach. We assessed the effects of frequent mating by analyzing changes in body weight, gonad mass, coloration, astaxanthin accumulation in the muscle and exoskeleton, and alterations in the muscle tissue structure. Additionally, RNA-seq, data-independent acquisition proteomics, and untargeted metabolomics were employed to analyze the gene expression profiles, protein spectra, and metabolic profiles of male *M. rosenbergii*. This study aims to elucidate the potential molecular mechanisms underlying the changes in the muscle energy metabolism in male prawns and provide insights into optimizing aquaculture management and improving growth performance and meat quality.

## 2. Results

### 2.1. Altered Physiological Status

**Figure 1:**
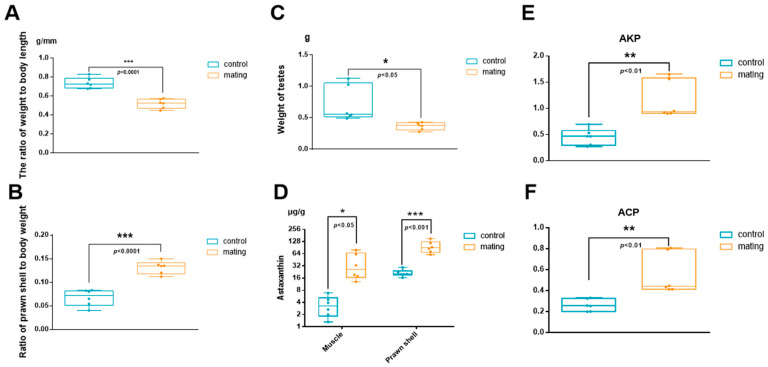
The effects of frequent mating on the body weight, gonads, astaxanthin content, and activity of enzymes in male *M. rosenbergii*. (**A**) The ratio of weight to body length. (**B**) The proportion of prawn shell weight. (**C**) Testis weight. (**D**) The astaxanthin content of muscles and prawn shells. (**E**) Alkaline phosphatase (AKP) activity in the hepatopancreas. (**F**) Acid phosphatase (ACP) activity in the hepatopancreas. *: *p* < 0.05; **: *p* < 0.01; ***: *p* < 0.001.

**Figure 2:**
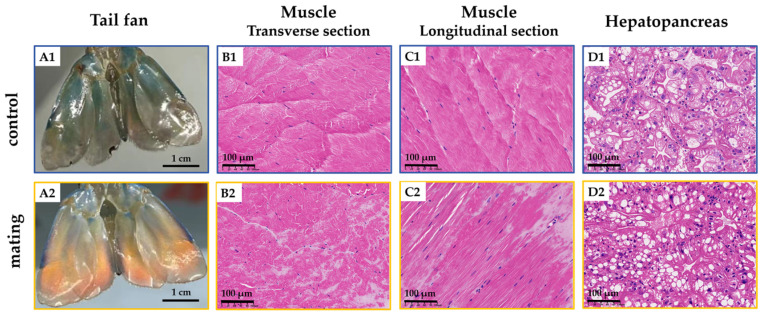
The effects of frequent mating on the coloration, hepatopancreas, and muscle tissue structure in male *M. rosenbergii*. Representative images of the tail fan color changes and H&E-stained sections of the muscle and hepatopancreas (×20); (**A1**) Tail fan in the control group; (**A2**) Tail fan in the mating group; (**B1**) Muscle transverse section in the control group; (**B2**) Muscle transverse section in the mating group; (**C1**) Muscle longitudinal section in the control group; (**C2**) Muscle longitudinal section in the mating group; (**D1**) Hepatopancreas sections in the control group; (**D2**) Hepatopancreas sections in the mating group. Scale bars in paraffin sections are 100 µm.

Male *M. rosenbergii* subjected to 30–40 consecutive mating events exhibited a significant reduction in body weight (Figure 1A), with body weight reaching only 68% of that observed in the control group. However, the proportion of shell weight to total body weight increased significantly and nearly doubled (Figure 1B). Testis mass decreased by 51% (Figure 1C), and notable alterations in body coloration were observed, particularly in the tail fan, which transitioned from blue-green to orange (Figure 2(A1,A2)). Additionally, astaxanthin accumulation in the exoskeleton and muscle tissue was 4.1 times (exoskeleton) and 2.6 times (muscle) greater than that in the control group (Figure 1D). The astaxanthin content was measured as 0.69 ± 0.24 µg/g in the muscle of the control group and 1.78 ± 0.97 µg/g in the muscle of the treatment group. In the prawn shell, the astaxanthin content was significantly higher, reaching 4.85 ± 1.78 µg/g in the control group and 19.69 ± 4.84 µg/g in the treatment group. Histological examination revealed muscle tissue degeneration, characterized by disorganized and dissolved myofiber alignment (Figure 2(B1,B2,C1,C2)). Histological sections revealed tightly connected tubular structures in the hepatopancreatic region with blurred boundaries (Figure 2(D1,D2)). In the hepatopancreas of the control group, the majority of lumens exhibited a stellate (star-like) shape. In contrast, in the mated group, the lumens were significantly narrowed, with some hepatic ducts almost completely collapsed and their lumens barely discernible. Within the same field of view, the number of fibrillar cells (F cells) and blister-like cells (B cells) increased, but the number of resorptive cells (R cells) did not change significantly (Figure 2(D1,D2)). Alkaline phosphatase (AKP) and acid phosphatase (ACP) activities were significantly increased (Figure 1E,F).

### 2.2. Transcriptome Analysis of Differentially Expressed Genes (DEGs)

**Figure 3:**
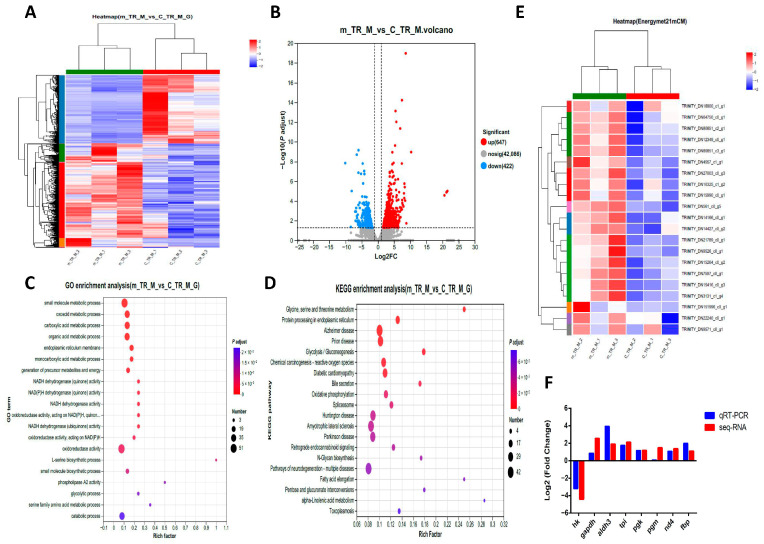
Effect of frequent mating on the global transcriptomic profiles of muscle tissues. (**A**) Heatmap of samples from the control and mating groups of *M. rosenbergii*. (**B**) Volcano plots showing significant DEGs in the muscles of the control and mating groups. Red and blue dots represent the upregulated and downregulated DEGs in frequently mated *M. rosenbergii* (*n* = 3), respectively. (**C**) The top 20 significantly enriched GO pathways for the DEGs. (**D**) The top 20 significantly enriched KEGG pathways for the DEGs. (**E**) Heatmap of the DEGs related to energy metabolism. (**F**) Comparison of the qRT-PCR expression and transcriptome results of 8 genes.

To investigate the impact of frequent mating behavior on muscle tissue, we examined the transcriptomes of muscle tissue from both normally mating and frequently mating male *M. rosenbergii*. Among the 43,155 identified transcripts, 1069 DEGs were identified, with 647 upregulated and 422 downregulated. The heatmap (Figure 3A) of the two groups demonstrated good reproducibility. Figure 3B presents a volcano plot of the DEGs, and Figure 3E shows a heatmap of 21 DEGs related to the energy metabolism between the two groups.

We determined the functional categories based on gene annotation (Figure 3C). The Gene Ontology (GO) enrichment terms of the DEGs included the metabolic processes of small molecules, carboxylic acids, organic acids, monocarboxylic acids, serine, and glycolysis, along with multiple GO entries related to the activity of NADH and NAD(P)H dehydrogenases. The 20 most significantly enriched KEGG pathways among these DEGs were glycolysis/gluconeogenesis; Alzheimer disease; Glycine; serine and threonine metabolism; protein processing in endoplasmic reticulum; Prion disease; Diabetic cardiomyopathy; chemical carcinogenesis—reactive oxygen species; Bile secretion; oxidative phosphorylation; Spliceosome; amyotrophic lateral sclerosis; Huntington’s disease; Parkinson’s disease; Retrograde endocannabinoid signaling; N-Glycan biosynthesis; pathways of neurodegeneration—multiple diseases; fatty acid elongation; Pentose and glucuronate interconversions; Valine; leucine and isoleucine degradation; and Toxoplasmosis (Figure 3D). We analyzed the expression of eight genes to confirm their expression profiles and validate the transcriptome data (Figure 3F). The results confirmed the reliability of our data and the transcriptome sequencing platform.

### 2.3. Proteomic Analysis

**Figure 4:**
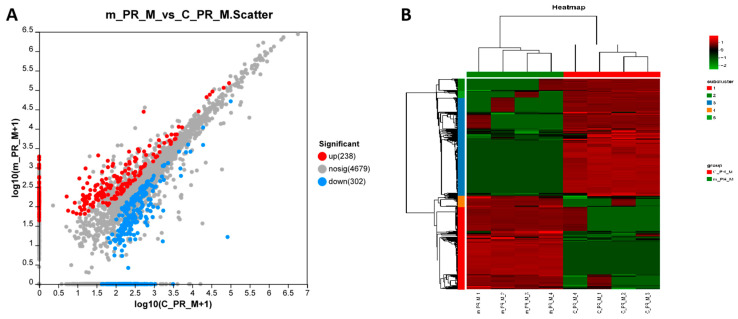
Effect of frequent mating on the proteomics of muscle tissues. (**A**) Scatter plot of differential proteins. (**B**) Cluster plot of differential proteins.

The muscle tissue of *M. rosenbergii* was analyzed using proteomics. As shown in Figure 4A, 5219 proteins were detected in the two different muscle tissues, with the expression levels of 540 proteins significantly differing (238 upregulated and 302 downregulated). Figure 4B shows the clustering pattern of the significantly different proteins, with good repeatability of the samples from different groups. Protein GSEA revealed that frequent mating males exhibited decreased abilities in the structural constituent of the ribosome, peptide biosynthetic process, and response to oxidative stress, among others. However, the lipase activity, proteasome complex, respiratory electron transport chain, and other aspects were increased (Table 1).

### 2.4. Metabolome Analysis

**Figure 5:**
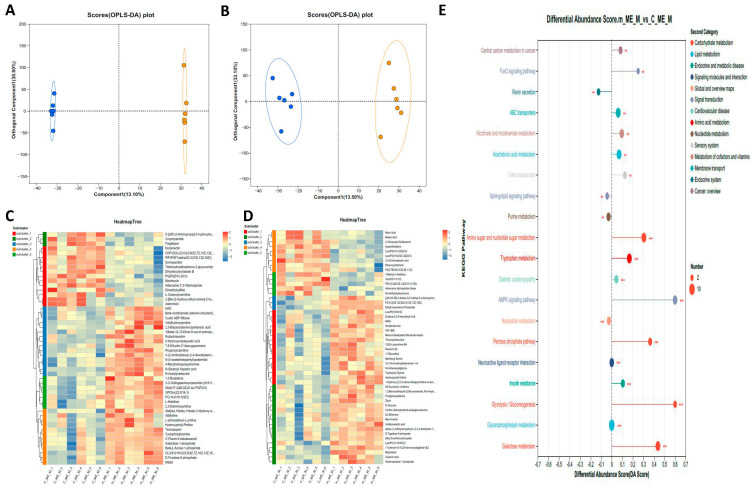
Effect of caponization on the global metabolomic profiles in muscle tissues. POS, positive ion mode; NEG, negative ion mode. (**A**,**B**) OPLS-DA scores showing significant differences between the caponized and control groups. (**C**,**D**) Heatmap analysis of the DEMs between the two groups. (**E**) Significantly altered metabolic pathways, **: *p* < 0.01; ***: *p* < 0.001. Larger dots indicate a greater number of differential metabolites in this pathway. The right side tended to be upregulated, and the left side tended to be downregulated. The longer the line segment, the stronger the tendency.

**Figure 6:**
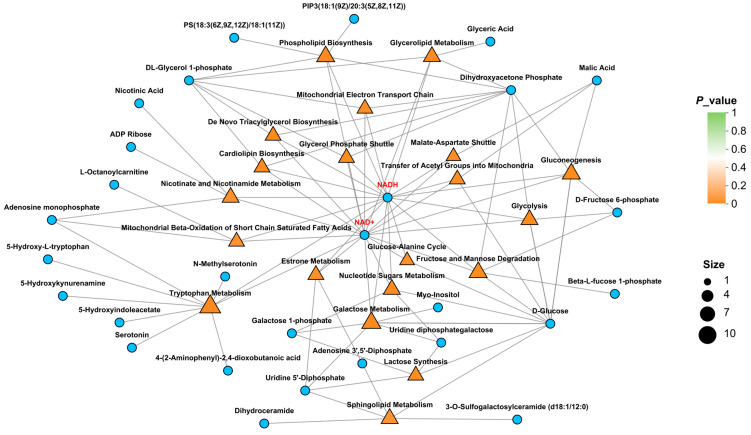
Metabolite Set Enrichment Analysis.

We obtained muscle tissue metabolomic data from male prawns to further elucidate the effect of frequent mating on energy metabolites. A total of 220 DEMs were identified, with 96 upregulated and 124 downregulated. Of these, 54 DEMs showed positive regulation and 91 DEMs showed negative regulation. Orthogonal Partial Least Squares Discriminant Analysis (OPLS-DA) revealed significant differences between the two groups in both positive and negative ion modes (Figure 5A,B). The heatmaps of the top 50 DEMs in both the positive and negative modes are shown in Figure 5C and Figure 5D, respectively. Figure 5E shows the top 20 KEGG-enriched pathways for the DEMs. Among these pathways, most showed a trend of upregulation, with several prominent pathways related to glucose metabolism. The MSEA results (Figure 6) showed that the differential metabolites were enriched in pathways such as glycolysis, fructose and mannose degradation, galactose metabolism, and glycerolipid metabolism. Notably, key metabolites—glucose and NAD+—were significantly upregulated. Additionally, serotonin (5-HT) was identified in the network graph, and its content was significantly increased (Table A1).

### 2.5. Multi-Omics Correlation Analysis

**Figure 7:**
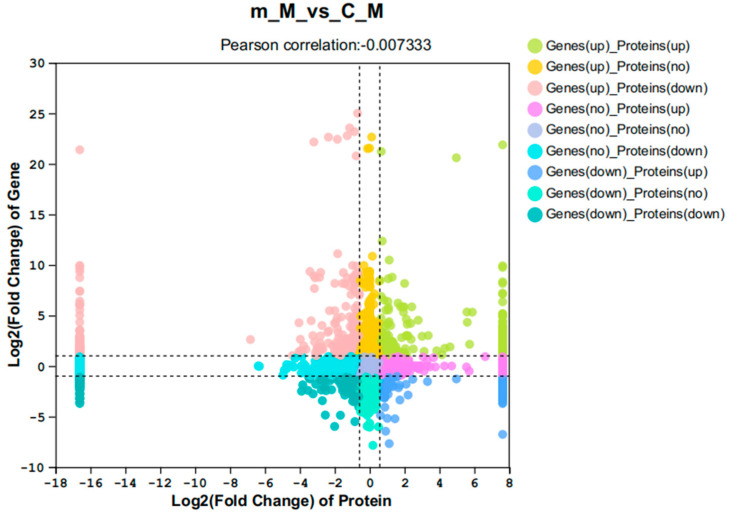
Nine-quadrant plots for combined analysis of transcriptome and proteome.

Using the combined analysis of the transcriptome and proteome, Figure 7 shows a nine-quadrant diagram, where a total of 117 significant differences were observed at both the transcript and protein levels. Among them, 32 were upregulated in both levels, 33 were downregulated in both levels, and 52 exhibited opposite expression patterns (Figure 7). The transcription and protein levels of fructose bisphosphate aldolase-like (aldo) and the V-type proton ATPase proteolipid subunit (atp6l) were significantly increased in frequently mating males. Both the transcriptional and protein levels of nuclear RNA export factor 1-like (nxf) and the unconventional myosin-IXa-like isoform X1 (myo9) were significantly decreased (Table A2).

**Figure 8:**
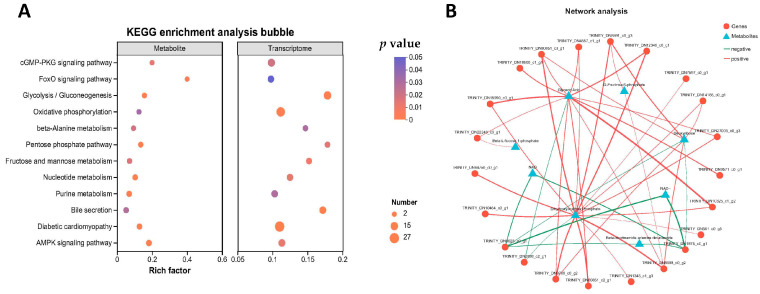
Results of combined analysis of transcriptome and metabolome. (**A**) KEGG enrichment pathways. (**B**) Correlation network analysis, where triangles represent metabolites, circles represent genes, lines represent correlation coefficients, red lines represent positive correlations, and green lines represent negative correlations.

The results of the combined transcriptome and metabolome analysis revealed that a total of 12 KEGG pathways were significantly enriched (Figure 8A). These pathways included glycolysis/gluconeogenesis, the Pentose Phosphate Pathway, oxidative phosphorylation, and galactose metabolism. Network analysis of transcripts and metabolites in these four pathways identified glyceric acid and dihydroxyacetone phosphate as the key metabolites (Figure 8B).

## 3. Discussion

In aquaculture, cultivating high-quality seedlings is crucial for success [18]. High-quality broodstock form the foundation is crucial for superior seedling cultivation, with male prawns playing a particularly significant role as providers of male gametes. These males typically mate with multiple females at a ratio of 1:3 to 1:7 [19,20]. The quality of male prawns is influenced by factors such as stocking density, feed composition, light cycles, and water environment [2,18,21].

In the course of long-term mating experiments, it was observed that some male prawns exhibited color changes after frequent mating. These males subsequently demonstrated a substantial decline in mating capability, with some even perishing. Male prawns from different families may have differences in genetic ability. For the family used in this study, the onset of these color changes typically occurred subsequent to 30–40 consecutive mating events. We sought to compare normal-phenotype males with those subjected to frequent mating. The results indicated that frequent mating had a significant impact on the physiological condition of male prawns, including a notable decrease in body weight, reduced gonad weight, muscle fiber dissolution and disorganization in muscle tissues, and hepatopancreas damage, as well as upregulation of the activities of hepatopancreas AKP and ACP and two immune-related enzymes [22]. Conversely, the proportion of shell weight increased, and the carotenoid content in both the shell and muscle tissues increased significantly. It is noteworthy that astaxanthin, the primary source of which is feed and algae in the water [23], exhibited a marked increase. The observations revealed that the males engaged in frequent mating and exhibited a marked tendency to guard the females, a behavior that could have a significant impact on their feeding patterns. Male animals expend a lot of energy during mating [24]. It is hypothesized that frequent mating depletes energy reserves in male prawns, resulting in excessive energy supply demands on the hepatopancreas, muscle tissues utilizing their own proteins for energy, and hindering shell regeneration to prioritize energy for reproductive behavior.

To investigate the impact of frequent mating on muscle tissue, we employed a multi-omics approach, including transcriptomics, proteomics, and metabolomics. Transcriptomic analysis revealed significant differences in gene expression related to carbohydrate and amino acid metabolism, with notable enrichment in pathways associated with degenerative muscle diseases (e.g., amyotrophic lateral sclerosis, Huntington’s disease, and Parkinson’s disease). Genes associated with energy metabolism (e.g., *gapdh*, *tpi*, *pgk*, and *fbp*) were upregulated, while hk2 was downregulated, potentially due to insulin resistance [25]. Despite the limitations of the proteomic data, GSEA analysis identified a decline in the ribosomal structure and peptide biosynthesis capacity, accompanied by a reduced oxidative stress response. Conversely, the expression of proteins involved in energy supply, such as lipase activity, proteasome complexes, and components of the respiratory electron transport chain, increased, suggesting that muscle proteins may play a key role in energy supply. Glucose is well known as one of the most important energy sources for cells, providing energy for various cellular processes [26]. NAD+ (nicotinamide adenine dinucleotide) and NADH (reduced nicotinamide adenine dinucleotide) are key coenzymes in cellular metabolism, involved in ATP generation and essential for cellular activities [27]. Metabolomics revealed that differential metabolites were enriched in pathways such as glycolysis, fructose, and mannose degradation; galactose metabolism; and glycerolipid metabolism, with glucose and NAD+ showing significant upregulation. A significant increase in the serotonin content was detected in the muscle tissue of frequent-mating M. rosenbergii males. The serotonin system, widely present in the central nervous system and spinal cord, regulates behavior through a variety of receptor subtypes [5]. Mating behavior involves complex muscle activity, which is regulated by serotonin [28].

The *aldo* gene, responsible for encoding fructose-1,6-bisphosphate aldolase—an essential enzyme in glycolysis [29]—and the *atp6l* gene, encoding the vacuolar-type proton ATPase V0 subunit C [30], both demonstrated significantly higher expression levels at both the transcript and protein levels in the muscles of males engaged in frequent mating. Conversely, the *nxf1* gene, coding for a key mRNA export protein [31], and the *myo9* gene, involved in cytoskeletal dynamics, cell migration, and signaling [32], exhibited substantial downregulation at both the transcript and protein levels. Integrated transcriptomic and metabolomic analysis highlighted energy metabolism pathways, particularly those related to carbohydrate and lipid metabolism, as significantly enriched. Network analysis suggested that glyceric acid and dihydroxyacetone phosphate (DHAP) were crucial metabolites. Glyceric acid plays a central role in cellular metabolism, particularly in glycerol and sugar metabolism, while DHAP bridges carbohydrate and lipid metabolism [33]. The upregulation of these metabolites may indicate that lipids in the muscles of frequent-mating males are predominantly utilized for energy.

HK2 is a key enzyme in glycolysis [34]. As hepatopancreatic damage worsens, muscle cells enter an insulin-resistant state, inhibiting the transcription of the *hk2* gene. This, in turn, leads to the breakdown of muscle proteins and lipids for energy, resulting in muscle cell damage. In less severe cases, this damage affects mating quality, but in more severe cases, it can lead to mortality. Therefore, regulating the frequency of male prawn usage during breeding cycles is vital.

Furthermore, *M. rosenbergii* males exhibit superior growth characteristics compared to females [2], and by prolonging the rearing time, individual larger prawns are obtained. Monosex breeding technology for *M. rosenbergii* has been highly developed [2,35]. To mitigate energy loss and muscle degradation caused by mating, it is recommended that males and females be segregated into separate ponds.

## 4. Materials and Methods

### 4.1. Animals and Experiment Design

*M. rosenbergii* were hatched in April 2023 in Changxing, China. After approximately 13 months of rearing, individuals with an average size of 14.7 ± 0.6 cm were obtained. All the prawns were from the same family. The samples consisted of male *M. rosenbergii* in two distinct physiological states. The two groups of *M. rosenbergii* are described as follows:

Control group, denoted as ‘C’: Individuals exhibited bright bluish-blue coloration, a shiny exoskeleton, high reactivity, and normal feeding behavior. They were raised for a month with females that were at an immature stage of ovarian development in order to avoid mating.

Treatment group (frequent-mating group, denoted as ‘m’): The female *M. rosenbergii* with ovaries full of the entire cephalothorax were put in and mated with the males. After the females acquired eggs, they were transferred, and new females were introduced to facilitate frequent mating. The copulation frequency of each male was calculated based on the number of females successfully fertilized. Within a month, after 30 to 40 matings, the males exhibited poor exoskeletal coloration, with individuals displaying an orange-blue body color, and some even showing a red tail fan. In addition, we found that at the corner of the breeding pond, males guard females and prepare for mating. During this period, males usually do not leave to feed, unless the feed happens to be in their vicinity.

### 4.2. Determination of Astaxanthin Content

The shell and muscle of *M. rosenbergii* were carefully separated. The muscle tissue was weighed and subjected to freeze-drying until no further weight reduction was observed. The final dry weight was recorded, and the muscle water content was subsequently calculated. A 1.0 g sample was homogenized with 10 mL of acetone. After sonication and centrifugation at 4 °C, 2 mL of the supernatant was collected. To this, 3 mL of a 0.02 mol/L NaOH solution (prepared in methanol) was added and mixed thoroughly. Following a 12 h incubation at 4 °C, 200 mg of NaHSO_4_ was introduced. The supernatant was then filtered and analyzed using a Waters 2695 HPLC system. The astaxanthin standards used in the experiments were sourced from Shanghai Yuanye Co., Ltd. (Shanghai, China). The astaxanthin standard was precisely weighed, dissolved in dichloromethane, and prepared into 5–6 standard solutions with different mass concentrations. The peak area of each standard solution was detected according to the above chromatographic conditions. The peak area was taken as the ordinate and the concentration as the abscissa, and the standard curve, linear range, and correlation coefficient of astaxanthin were calculated. The experimental temperature was controlled at 30 °C, with the sample flow rate: 1 mL/min, injection volume: 10 µL, and mobile phase: methanol.

### 4.3. Measurement of Alkaline Phosphatase (AKP) and Acid Phosphatase (ACP) Activity

The alkaline phosphatase (AKP) and acid phosphatase (ACP) activities were measured using kits (A059-1-1, A060-1-1) from Nanjing Jiancheng Biological Co., Ltd., (Nanjing, China). The experiments were carried out according to the instructions.

### 4.4. Histological Observation

The hematoxylin and eosin (H&E) staining of the hepatopancreas and muscle was performed as follows: the hepatopancreas and trunk muscle tissues were collected and washed three times with phosphate-buffered saline (PBS), fixed in Bouin’s solution for less than 24 h, and then transferred to 4% paraformaldehyde. It is important to note that hepatopancreas was washed before sampling, whereas muscle tissue was washed after. After dehydration, the tissues were embedded in paraffin, sectioned into 4 µm slices, and stained with hematoxylin and eosin. Histological changes in the muscle tissue were observed under a microscope.

### 4.5. Sequencing and Analysis

Sequencing of the transcriptome, proteome, and metabolome were completed by Shanghai Meiji Biomedical Technology Co., Ltd. (Shanghai, China). The data were analyzed on the online tool of the Majorbio Cloud Platform https://cloud.majorbio.com/page/tools/ (accessed on 5 November 2024) [36].

RNA sequencing: The total RNA of *M. rosenbergii* was prepared from the carapaces using Trizol method (Invitrogen, Waltham, MA, USA). Using magnetic beads coated with Oligo(dT), mRNA was enriched from the sample. The mRNA was then randomly fragmented into segments of approximately 300 bp. Reverse transcription was performed using reverse transcriptase to synthesize complementary DNA (cDNA), followed by second-strand synthesis to form a stable double-stranded structure. Adapter sequences were ligated to the ends of the DNA fragments. The adapter-ligated products were purified and size-selected, and the selected fragments were amplified via PCR. The final library was purified and sequenced on the NovaSeq X Plus platform. Genes were classified as the differentially expressed genes (DEGs) if false discovery rate ≤ 0.05 and |log2 (fold-change)| ≥ 1. Each group contained three replicates.

Proteome sequencing: The frozen muscle sample was retrieved and an appropriate amount of protein lysis buffer was added (8 M urea + 1% SDS, containing protease inhibitors) for homogenization. The protein concentration was measured and 100 µg of the protein sample was taken for trypsin digestion (37 °C, 12 h) to obtain peptide fragments. Equal amounts of peptides were dissolved in MS loading buffer for DIA analysis. Peptide separation was performed using a Vanquish Neo chromatography system (Thermo, Waltham, MA, USA), with a chromatographic runtime of 8 min. Data acquisition was carried out using Thermo Xcalibur 4.7 software (Thermo, Waltham, MA, USA). The Orbitrap Astral mass spectrometer (Thermo) operated in a DIA mode for MS analysis, with a scan range of 100–1700 m/z. Proteins were judged to be significantly regulated (*p* ≤ 0.05) for multiplicity changes ≥ 1.5 (upregulated) or ≤ 0.67 (downregulated). Gene Set Enrichment Analysis (GSEA) was conducted on all proteins. Each group contained four replicates.

Metabolomic sequencing: A total of 50 mg of the solid sample was weighed and 400 µL of extraction solution was added (methanol–water = 4:1, *v*/*v*). The sample was ground in a cryogenic tissue grinder for 6 min at −10 °C and 50 Hz. Low-temperature ultrasonic extraction was performed for 30 min at 5 °C and 40 kHz. The sample was kept at −20 °C for 30 min and then centrifuged at 4 °C and 13,000× *g* for 15 min. The supernatant was collected as the sample for LC-MS/MS analysis. The sample was analyzed using a Thermo Fisher Scientific UHPLC-Q Exactive HF-X system for LC-MS/MS analysis. Differential metabolites were identified based on variable importance in projection (VIP) values derived from the Orthogonal Partial Least Squares Discriminant Analysis (OPLS-DA) model, with metabolites having a VIP > 1 and a *p*-value < 0.05, determined by Student’s *t*-test, considered significantly different. Each group contained six replicates.

### 4.6. Real-Time Quantitative PCR (qPCR) Analysis of Gene Expression

The total qPCR reaction system was composed of 20 µL, containing 1 µL of cDNA (5 ng/µL), 0.4 µL of each primer pair (Table 2), and 10 µL of 2× ChamQ Blue Universal SYBR qPCR Master Mix (Vazyme, Nanjing, China). The reaction mixture was added to a 96-well plate (NEST, China) in a low-light environment, followed by brief centrifugation. qPCR was performed using a fluorescent quantitative PCR system (Roche LightCycler 96) under the following conditions: an initial denaturation at 95 °C for 2 min, followed by 45 cycles of 95 °C for 10 s and 60 °C for 30 s. The relative expression levels of target genes were calculated using the 2^−∆∆Ct^ method, with reference genes as internal controls [37].

### 4.7. Statiscical Analyses

All data are expressed as mean ± SE and drawn using GraphPad Prism 6.01 (GraphPad Software, San Diego, CA, USA). The transcriptome analysis contained three replicates, the proteome contained four replicates, and the metabolome analysis contained six replicates. Data were analyzed by multiple *t* test, and statistical significance was set at *p* < 0.05.

## 5. Conclusions

In this study, we comprehensively evaluated the effects of frequent mating on muscle mass in male *M. rosenbergii*. The results showed that frequent mating led to significant changes in gene expression, protein translation, and metabolism in male *M. rosenbergii*. A total of 1069 DEGs and 385 DEMs were significantly enriched in pathways related to energy metabolism and degenerative diseases. Based on the annotation of 5219 proteins, the GSEA results suggested that the proteins and lipids in muscle tissues provide energy for frequently mating *M. rosenbergii*. In conclusion, we infer that frequent mating behaviors compel males to allocate significant energy and resources toward reproductive activities, organ development, and germ cell production. Excessive mating leads to protein degradation in muscle cells to meet energy demands, ultimately causing structural disorganization of muscle tissue, while serotonin elevation and *hk2* downregulation reflect neuromuscular regulatory adaptations to mating stress. The findings of this study provide valuable insights for managing male health during the breeding process of *M. rosenbergii*.

## Figures and Tables

**Table 1:** Results of protein aggregation GSEA enrichment analysis.

GO Description	NES	*p*-Value	Lead|Size
structural constituent of ribosome	−2.15175	0	78|89
structural molecule activity	−2.01916	0	117|162
peptide biosynthetic process	−1.90523	0	74|88
ribosome	−1.85941	0	60|76
translation	−1.91465	0	73|86
ribosomal subunit	−1.86037	0.00173	30|34
amide biosynthetic process	−1.87565	0	78|98
cellular response to chemical stimulus	−1.80275	0.00169	7|19
ribonucleoprotein complex	−1.79437	0	108|189
peptide metabolic process	−1.80315	0	75|95
cellular protein metabolic process	−1.81616	0	89|122
microtubule cytoskeleton organization	−1.78661	0.00338	9|19
response to chemical	−1.80472	0.00173	13|35
cellular amide metabolic process	−1.76313	0	77|121
response to oxidative stress	−1.75177	0.0049	12|25
cysteine-type peptidase activity	−1.68567	0.00333	13|53
aminopeptidase activity	−1.69007	0.00671	7|19
hydrolase activity, hydrolyzing O-glycosyl compounds	−1.64148	0.00326	17|58
response to organic substance	−1.64371	0.00858	8|24
endopeptidase complex	1.73946	0.00472	16|20
respiratory chain complex	1.72275	0.01256	19|21
proteasome complex	1.71102	0.01946	16|20
peroxidase activity	−1.62955	0.01544	8|19
transferase activity, transferring sulfur-containing groups	1.68865	0.00515	11|21
carboxylic acid binding	1.7413	0.01446	2|17
respiratory electron transport chain	1.75704	0.00699	16|20
generation of precursor metabolites and energy	1.76887	0.00272	33|55
lipase activity	1.59569	0.03373	2|18
mitochondrial protein complex	1.58684	0.01961	25|40
organic acid binding	1.65345	0.00948	2|19
whole membrane	1.59702	0.00532	14|49
tricarboxylic acid cycle	1.63374	0.02232	12|18
proteasomal protein catabolic process	1.60833	0.01887	13|17
extracellular region part	1.5984	0.00543	10|85
inner mitochondrial membrane protein complex	1.63885	0.02	23|35
electron transport chain	1.81405	0	19|23
peptidase complex	1.60913	0.02353	17|24
citrate metabolic process	1.61652	0.03	12|18
ubiquitin-dependent protein catabolic process	1.77131	0.00726	22|33
sulfotransferase activity	1.83354	0	11|18

Notes: 1. GO Description: GO protein set description; 2. NES: standardized enrichment score; positive values represent higher expression overall in the experimental group and negative values represent higher expression in the control group; 3. *p*-value: a statistical test was performed for the enrichment scores obtained; 4. Lead|Size: the protein member that contributed most to the enrichment score | the total number of proteins under the protein set.

**Table 2:** Primers used for qRT-PCR.

Gene	Function	Primer Pairs (5′-3′)	Accession Number
*aldh3*	To convert aldehydes to the corresponding carboxylic acids.	F: TGTACGTCGAGAACGAGCAG R: CGCCCATAACGAGAACCACT	XM_067104159.1
*fbp*	It catalyzes the conversion of fructose-1, 6-diphosphate to fructose6-phosphate, which is the rate-limiting step in the gluconeogenesis pathway.	F: GCAGGTCGAGTACGAGGATG R: ATGATGGGCATCCAGACAGC	XM_067125270.1
*hk*	It catalyzes the conversion of glucose to glucose-6-phosphate, the first step in glycolysis.	F: CTCCAAACGCTCCCCATTCT R: TACAACTGGCACGCTCATGT	XM_067093332.1
*nd4*	It is involved in the electron transport chain and is responsible for the transfer of electrons from NADH to ubiquinone.	F: GCCCAGGCTTTCAGTCATCT R: AGACACCTGCACAGCTAACC	XM_067108213.1
*pgk*	It catalyzes the conversion of 1, 3-diphosphoglycerate to 3-phosphoglycerate, along with the generation of ATP, and is a key enzyme in the glycolytic and gluconeogenic pathways.	F: GGATGGGCCTTGATTGTGGA R: CGTGCATCCTTTGGTTGTGG	XM_067098515.1
*pgm*	It catalyzes the conversion between glucose-1-phosphate and glucose-6-phosphate.	F: ATTCCTGGGTGCCGATCTTG R: GGTGGCCCTAATGCTGACTT	XM_067133232.1
*tpi*	It catalyzes the interconversion between dihydroxyacetone phosphate and glyceraldehyde-3-phosphate.	F: CAAATGAAGGCGCTGGTTCC R: CACCTGGGCACTGACGTTAT	XM_067106417.1
*gapdh*	It catalyzes the oxidation and phosphorylation of glyceraldehyde-3-phosphate and is a key enzyme in glycolysis and gluconeogenesis pathways.	F: TGAAGCCCGAGAACATTCCATG R: GTTCACGCCGCAGACGAACATG	XM_067126079.1
*rps18*	It encodes the ribosomal protein S18, a component of the small ribosomal subunit.	F: TACCTACGACCCACACCCTT R: TATCAACGCACCGCCAAGAT	XM_067093923.1

## Data Availability

All data directly relevant to this paper are available in the main text or Appendix A.

## Abbreviations

*M. rosenbergii*	*Macrobrachium rosenbergii*
KEGG	Kyoto Encyclopedia of Genes and Genomes
GO	Gene Ontology
DEGs	differentially expressed genes
DEPs	differentially expressed proteins
DEMs	differentially expressed metabolites
H&E	hematoxylin and eosin
GSEA	Gene Set Enrichment Analysis
MSEA	Metabolite Set Enrichment Analysis
*aldh3*	aldehyde dehydrogenase 3 family member A1
*fbp*	fructose-1,6-bisphosphatase
*hk*	hexokinase
*nd4*	NADH dehydrogenase subunit 4
*pgk*	phosphoglycerate kinase
*pgm*	phosphoglucomutase
*tpi*	triosephosphate isomerase
*gapdh*	glyceraldehyde-3-phosphate dehydrogenase
*rps18*	ribosomal protein S18

## Appendix A

**Table A1:** Several notable differential metabolites.

Metabolite	*p*_Value	Log2FC(m_ME_M/C_ME_M)	Significant	Regulate
NAD	0.008888	0.0599	yes	up
NAD+	0.01724	0.0613	yes	up
Serotonin	0.006341	0.3161	yes	up
N-Methylserotonin	0.04121	0.6014	yes	up
Dihydroxyacetone Phosphate	0.001331	0.0909	yes	up
Glyceric Acid	0.02769	0.0986	yes	up
D-Glucose	0.0382	0.0475	yes	up

**Table A2:** Significantly upregulated and downregulated genes in both transcriptome and proteome.

NR_Description	Gene(log2FC)	Protein(log2FC)
fructose-bisphosphate aldolase-like	2.49	1.243
transketolase-like protein 2	3.918	7.624
uncharacterized protein LOC113813620	2.732	7.624
uncharacterized protein LOC113812620	2.636	2.378
C-reactive protein 1.1-like	3.063	7.624
antilipopolysaccharide factor	3.732	7.624
RNA-binding protein with serine-rich domain 1-A	1.892	7.624
X-ray repair cross-complementing protein 5-like isoform X3	2.491	7.624
uncharacterized protein LOC122257736	2.265	7.624
V-type proton ATPase proteolipid subunit	1.345	1.604
mid1-interacting protein 1A-like isoform X2	4.131	2.154
peroxisomal membrane protein PMP34-like	3.208	7.624
vacuolar fusion protein MON1 homolog A-like isoform X2	1.701	7.624
talin-2-like isoform X1	1.697	7.624
sphingomyelin phosphodiesterase 4-like	1.676	7.624
serine protease inhibitor 88Ea-like	3.591	7.624
hypothetical protein C7M84_025254	2.971	7.624
venom protease-like	2.626	7.624
F-box-like/WD repeat-containing protein TBL1XR1 isoform X2	1.676	7.624
coiled-coil domain-containing protein 56	1.776	7.624
very low-density lipoprotein receptor-like	2.115	7.624
metaxin-1-like isoform X1	1.852	7.624
protein PTCD3 homolog, mitochondrial-like	2.368	7.624
uncharacterized protein LOC113804706 isoform X2	1.685	1.483
epoxide hydrolase 4-like	2.559	7.624
acylglycerol kinase, mitochondrial-like	1.865	7.624
uncharacterized protein LOC121857022 isoform X12	2.965	0.9386
cytosolic non-specific dipeptidase-like isoform X2	2.276	7.624
uncharacterized protein LOC123770307	4.642	2.102
protein qua-1-like	−1.466	−16.61
fibrillin-3-like isoform X2	−2.351	−2.179
iodotyrosine deiodinase 1-like	−2.145	−16.61
uncharacterized protein LOC123763680	−2.486	−0.679
periodic tryptophan protein 1 homolog	−1.836	−1.432
unconventional myosin-IXa-like isoform X1	−2.054	−16.61
rab11 family-interacting protein 1-like	−2.002	−16.61
DnaJ C2	−2.371	−2.764
nuclear RNA export factor 1-like	−2.435	−1.548
serine/arginine repetitive matrix protein 2-like isoform X5	−1.819	−16.61
4′-phosphopantetheine phosphatase-like isoform X3	−1.479	−0.8173
synapse-associated protein 1	−1.284	−16.61
unnamed protein product	−4.851	−2.539
leucine-rich repeat protein SHOC-2-like isoform X1	−2.67	−16.61
uncharacterized protein LOC123773768	−2.068	−2.554
equilibrative nucleoside transporter 1-like isoform X3	−2.091	−16.61
monocarboxylate transporter 3-like isoform X1	−1.536	−1.413
endophilin-A-like isoform X5	−2.636	−0.9748
uncharacterized protein ZK1073.1-like isoform X3	−1.991	−0.7415
uncharacterized protein LOC113817296	−3.43	−2.708
troponin C, isotype gamma-like isoform X5	−5.5	−0.844
protein stum homolog	−2.843	−16.61
tyrosine-protein phosphatase non-receptor type 9-like isoform X3	−1.628	−1.585
soluble calcium-activated nucleotidase 1-like	−2.356	−16.61
golgin subfamily A member 2-like	−1.806	−16.61
protein quiver-like isoform X2	−2.448	−0.8967
probable phospholipid-transporting ATPase IM isoform X3	−1.542	−0.9456
1-phosphatidylinositol 4,5-bisphosphate phosphodiesterase delta-4-like	−1.787	−1.369
Arf-GAP domain and FG repeat-containing protein 1-like	−2.003	−0.737
uncharacterized protein LOC122248415 isoform X1	−1.684	−0.7171
low-quality protein: MOG-interacting and ectopic P-granules protein 1-like	−2.206	−16.61
crustacyanin-A1	−4.871	−1.673

Note: only the results annotated in the Non-Redundant Protein Sequence Database are shown in the table.
